# Comparison of three reconstruction techniques for medial column support loss in elderly patients with proximal humeral fractures

**DOI:** 10.3389/fmed.2026.1779683

**Published:** 2026-05-08

**Authors:** Jianchuan Wang, Qiwen Xue, Tienan Wang, Hongbin Hao, Yong Wang, Xiaowei Ma

**Affiliations:** 1 School of Mechanical Engineering, Dalian Jiaotong University, Shahekou, Dalian, China; 2 Department of Orthopedics, Affiliated Zhongshan Hospital of Dalia University, Dalian, China; 3 Dalian Medical University, Dalian, China

**Keywords:** front inner support, iliac bone graft, internal fixation of steel plate, proximal humeral fracture, reconstruction technique

## Abstract

**Objective:**

To compare the therapeutic outcomes of lateral locking plate combined with anterior medial support plate, locking plate combined with autogenous iliac bone graft, and simple lateral locking plate in the reconstruction of medial column support loss in elderly patients with proximal humeral fractures.

**Methods:**

A retrospective cohort study was conducted to analyze the data of 51 elderly patients with proximal humeral fractures who were admitted to the Orthopedics Department of Zhongshan Hospital affiliated with Dalian University from February 2016 to May 2020 and suffered from medial column support deficiency. 16 males and 35 females; Age (62.4 ± 10.2) years old. 17 cases were treated with lateral locking steel plate combined with anterior medial support steel plate (double plate group), 17 cases were treated with locking steel plate combined with autogenous iliac bone graft (bone graft group), and 17 cases were treated with lateral PHILOS steel plate alone (steel plate group). Compare the hospitalization time, intraoperative blood loss, fracture healing time, neck shaft angle, humeral head loss height, and shoulder joint range of motion (flexion, abduction, external rotation, extension) among three groups of patients at the last follow-up. At the last follow-up, use pain visual analog scale (VAS), American Society of Shoulder and Elbow Surgeons (ASES), disability of the arm, shoulder, and hand (DASH) score, and shoulder joint function Constant Murley score to observe the incidence of complications.

**Results:**

All 51 patients were followed up for 12–26 months (mean 16.8 ± 2.6 months). There was no statistically significant difference in preoperative general information among the three groups of patients (*P* > 0.05), indicating comparability. The bleeding volume of the three groups was 450.6 ± 13.7 ml, 380.5 ± 15.3 ml, and 280.2 ± 10.3 ml, respectively, and the differences between the two groups were statistically significant (*P* < 0.05). There was no statistically significant difference in hospitalization time and fracture healing time among the three groups of patients (*P* > 0.05); at the last follow-up, the neck shaft angles were 132.1° ± 9.8°, 125.3° ± 5.4°, and 120.1° ± 7.5°, respectively, with statistically significant differences between the two groups (*P* < 0.05). At the last follow-up, the height of humeral head loss was 1.32 mm ± 0.33 mm, 2.04 mm ± 0.56 mm, and 3.12 mm ± 0.85 mm, respectively, with statistically significant differences between the two groups (*P* < 0.05). At the last follow-up, the range of motion for shoulder flexion was 158.6° ± 12.8°, 149.3° ± 10.5°, and 140.2° ± 11.3°, respectively, with statistically significant differences between the two groups (*P* < 0.05); The outreach activity degrees were 148.2° ± 10.4°, 140.3° ± 11.5°, and 134.3° ± 12.6°, respectively, and the differences between the two groups were statistically significant (*P* < 0.05); the external rotation range of motion was 41.2° ± 7.8°, 40.5° ± 6.1°, 39.8° ± 7.4°, and the backward extension range of motion was 36.6° ± 1.2°, 35.8° ± 1.0°, 35.2° ± 1.4°, respectively. There was no statistically significant difference in the comparison between the three groups (*P* > 0.05). At the last follow-up, there was no statistically significant difference in VAS score, ASES score, DASH score, and Constant Murley score among the three groups (*P* > 0.05). There were 1, 2, and 3 patients with complications in each of the three groups, respectively, with no statistically significant difference observed among the groups (*P* > 0.05).

**Conclusion:**

For complex fractures of the proximal humerus in elderly patients with missing medial column support, all three reconstruction techniques can effectively reconstruct the stability of the medial column. However, the combination of a lateral locking plate and an anteromedial support plate (dual plating, Group A) demonstrated superior outcomes in maintaining the postoperative neck shaft angle, prevent loss of humeral head height, improve shoulder joint mobility, promote functional recovery, and have the advantage of fewer complications. However, this technique is also associated with disadvantages such as extensive soft tissue dissection and a relatively complex surgical procedure. Treatment selection should be individualized based on patient factors and surgeon experience.

## Introduction

Proximal humeral fracture is a common orthopedic injury. It is the third most common fracture among the elderly, following hip fractures and distal radius fractures, with an increasing incidence rate (1). Proximal humeral fractures account for about 5%−9% of all fractures in adults, and are more common in elderly patients with osteoporosis, especially women. In this age group where osteoporosis is common, even simple injuries from falls can lead to serious fractures. The incidence rate of proximal humeral fractures will further increase as the population structure changes, leading to social aging, It is expected to increase three-fold in the next 30 years (2, 3). Although the incidence rate is getting higher and higher, determining the best method to treat these fractures is still a dynamic research field, because many external factors play an important role in the treatment and results of these injuries, such as the patient's age, complications, activity level, fracture morphology and the complex anatomical structure around the shoulder joint. These factors not only increase the complexity of treatment decisions, but also are very important for determining the best treatment, functional recovery and imaging recovery of such fractures (4). In osteoporotic proximal humeral fractures, complex Neer three- and four-part fractures constitute a substantial proportion of cases in the elderly population. When treated non-operatively, these unstable fracture patterns are associated with higher risks of non-union, malunion, stiffness, and functional limitations, and many authors therefore recommend surgical fixation for such injuries. Although various surgical options are available, locking plate fixation has become one of the most commonly used techniques because it provides reliable mechanical stability in osteoporotic bone (5). However, there are also many postoperative problems, such as screw penetration, humeral head collapse, inversion deformity, humeral head necrosis and other complications. Many scholars believe that the lack of medial column support is one of the main risk factors for implant failure and complications (6, 7). Therefore, while using locking steel plates for fixation, the missing inner column should be reconstructed to increase clinical efficacy. Therefore, we conducted a retrospective cohort study to compare the clinical outcomes and complication rates among three techniques for reconstructing medial column support loss in elderly patients with proximal humeral fractures.

## Materials and methods

### Participants

This study was a retrospective cohort study that included 51 elderly patients with proximal humerus fractures and medial column support deficiency who were admitted to the Department of Orthopedic Trauma, Affiliated Zhongshan Hospital of Dalian University, between February 2016 and May 2020. Inclusion criteria: ① X-ray and CT diagnosis of Neer classification 3 and 4 partial fractures of the proximal humerus; ② Fresh closed proximal humeral fracture with radiographic evidence of medial column support loss: preoperative anteroposterior shoulder radiographs and CT three-dimensional reconstruction showing disruption or comminution of the medial humeral neck cortex, a reduced neck-shaft angle with varus malalignment compared with the contralateral side, and/or a metaphyseal defect beneath the humeral head indicating medial column deficiency; ③ Age predominantly ≥50 years, with a few patients aged ≥50 years who were diagnosed with osteoporosis by bone mineral density (BMD) testing and met the other inclusion criteria; ④ No previous significant shoulder pain, restricted movement, or MRI-confirmed chronic rotator cuff tears; ⑤ Informed consent and complete clinical follow-up data. Exclusion criteria: ① Patients with concurrent fractures or nerve or vascular injuries in other parts of the upper limb on the same side; ② Patients with pathological or open fractures; ③ Severe combined basic or weak constitution, unable to tolerate surgery; ④ History of ipsilateral shoulder joint rotator cuff injury or chronic pain; ⑤ Not willing to accept, incomplete clinical follow-up data.

All patients underwent anterior-lateral X-rays of both shoulder joints, CT and MRI examinations of the affected shoulders before the operation. CT and three-dimensional reconstruction confirmed the defect of the medial support bone of the proximal humerus. According to different treatment methods. A comparative analysis was performed between groups based on the treatment method received.

The study protocol was approved by the Ethics Committee of Zhongshan Hospital Affiliated to Dalian University (2020001), and written informed consent was obtained from all patients.

### Surgical method

The selection of surgical approach should be based on a comprehensive consideration of factors including patient age, bone quality, fracture type, and the extent of medial column involvement. In cases with significant medial cortical bone defects, a tendency for varus deformity, or severe metaphyseal comminution, priority should be given to the use of a lateral locking plate combined with an anteromedial buttress plate, or a locking plate combined with autologous iliac bone grafting, to restore medial column support. For patients with relatively intact medial cortex, less severe comminution, and fewer medial instability factors, isolated lateral locking plate fixation may be considered. The final surgical strategy is determined by an experienced upper limb orthopedic surgeon based on preoperative imaging assessment and intraoperative findings.

All three groups of patients who met the inclusion criteria were given general anesthesia. The patients were in a supine position. The shoulder on the surgical side was elevated. The surgical area and the site where the iliac bone was extracted were routinely disinfected. Surgical sheets were laid and transparent films were applied to the surgical area.

**Figure 1:**
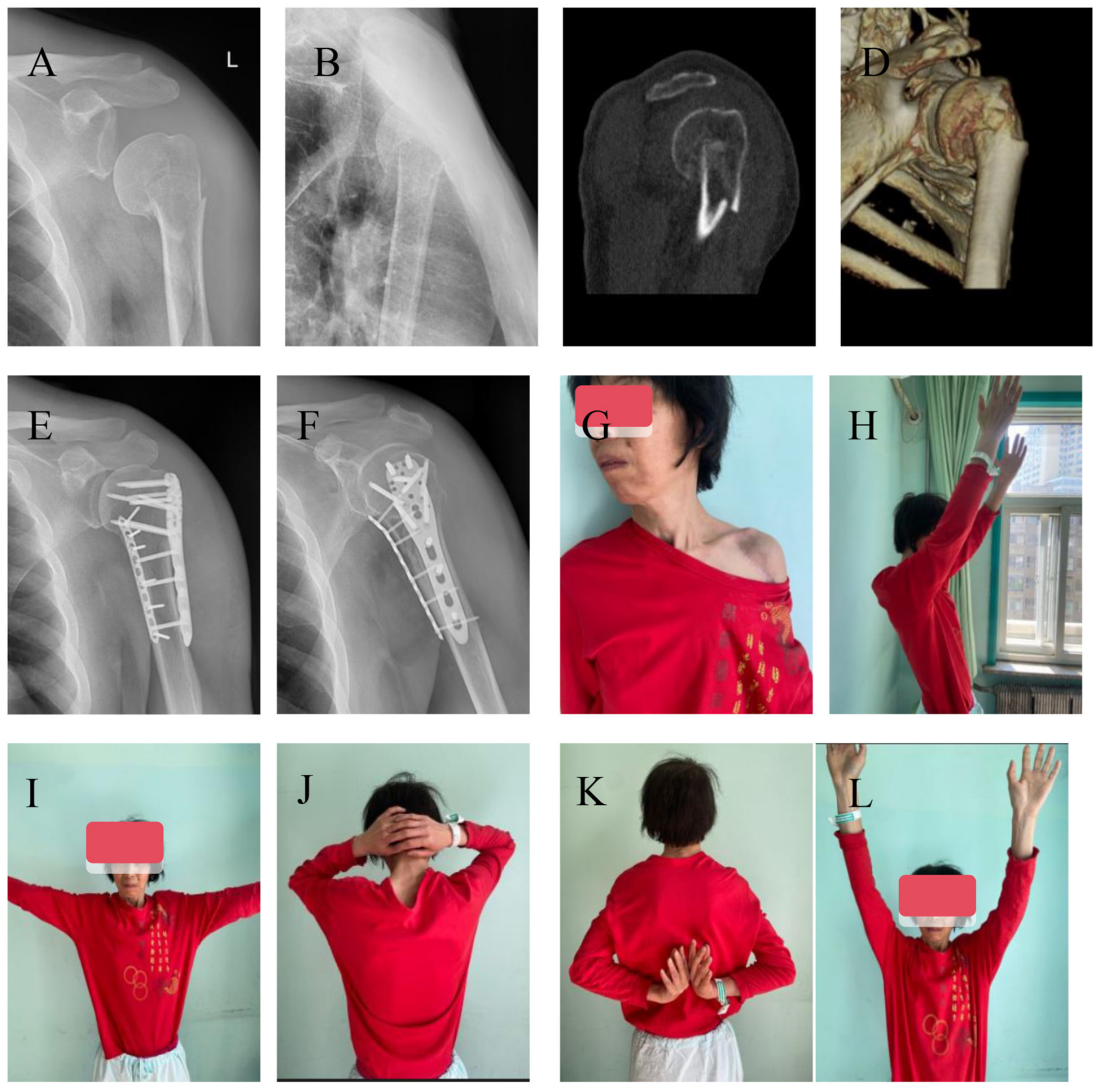
A 65-year-old female suffered a proximal humeral fracture due to a traffic accident with loss of the medial humeral tuberosity support. She was treated with double plate technique. The patient was followed up for 16.2 months. **(A, B)** X-ray anteroposterior and lateral views showed loss of the medial column support of the proximal humerus and varus deformity of the humeral head. **(C, D)** Sagittal and three-dimensional CT reconstruction showed loss of the medial column of the humeral tuberosity. **(E, F)** The last follow-up X-ray anteroposterior and lateral views showed anatomical reduction of the fracture, no displacement of the internal fixation, and fracture healing. **(G–L)** The appearance of the left shoulder after surgery and the recovery of shoulder joint flexion, abduction, external rotation and extension functions at the last follow-up were good.

The outer locking steel plate combined with the front inner side support steel plate (Double-board group):

The deltopectoral approach was adopted. The deltoid was pulled laterally, the cephalic vein was mobilized and retracted to expose it. The axillary nerve and the long head tendon of the biceps brachii were identified, tagged, and protected with saline-soaked gauze to fully expose the fracture site and protect the periosteum. The fracture was reduced under direct vision to ensure good fracture reduction. Temporary fixation with Kirschler's needle was used to maintain the reduction. Place the lateral locking plate 0.5 cm from the lateral edge of the biceps tendon and 0.5 cm from the lower edge of the large nodule. Screw in one cortical screw placed in the compression hole through the sliding hole of the locking plate for fixation. Confirm the height of the plate by X-ray under the fluoroscopy and fine-adjust it to a satisfactory position if necessary. Insert 6 to 8 locking screws into the proximal locking hole and screw in 3 locking screws. Remove the temporary fixing Kirschner pins and tension screws. The upper limb was then externally rotated to expose the lesser tuberosity. An anteromedial support plate was positioned approximately 0.5 cm medial to the biceps tendon groove, oriented approximately perpendicular to the lateral plate. The plate was temporarily fixed with a single locking screw. Re-examine the fluoroscopy to confirm the position of the plate. After confirming that the positions of the inner and lateral plates are appropriate, insert the locking screws in sequence. Move the shoulder joint to confirm that the plate is not impacted. Rinse the incision and place the surgical drain. Suture the incision layer by layer to close it, apply pressure bandaging with sterile cotton pads, and fix the shoulder joint with a 45° shoulder abduction brace (Typical cases are shown in Figure 1).

Locking steel plate combined with autologous iliac bone grafting (bone grafting group):

First, a complete iliac bone block was taken from the ipsilateral iliac bone, wrapped and protected with saline gauze. The deltoid—pectoralis major muscle space approach was adopted to protect the axillary nerve and the long head tendon of the biceps brachii. The proximal humeral fracture site was fully exposed. The terminally fractured hematoma and soft tissue were cleared. The spare iliac bone block was trimmed according to the degree of bone defect. The cortical surface of the iliac bone block was placed parallel to the medullary cavity of the brachial shaft, and the proximal end was pressed inward as much as possible. Fully support the humeral head, fill the space around the medullary cavity with cancellous bone, temporarily fix the iliac bone mass in the medullary cavity with Kreutz needle to prevent the bone mass from sinking, reduce the proximal humeral fracture mass under direct vision, fluoroscopy fluoroscopy confirmed good fracture reduction, 0.5 cm outside the internodal sulcus. A locking plate was placed on the outside 0.5 cm below the apex of the large nodule. One locking plate was temporarily fixed. The fluoroscopy underwent re-examination to confirm the position of the plate and the satisfaction of fracture reduction. The locking screws were screwed in successively. The shoulder joint was moved to confirm that the plate had no impact. The incision was rinsed, a surgical drain was placed, and the incision was sutured layer by layer to close. Sterile cotton pads were used for compression bandage, and the shoulder joint was abducted at 45° and fixed with a brace.

Only the outer PHILOS steel plate is adopted (Steel plate group): the surgical approach and operation process of simply using the lateral PHILOS plate were the same as those of the lateral plate fixation scheme in the double-plate group.

### Postoperative management and outcome assessment

The patients in the three groups were protected by 45° shoulder abduction braces after the operation. Passive shoulder range of motion exercises were initiated at 2 weeks postoperatively to avoid shoulder joint adhesions. The external fixation braces were removed in the third week after the operation, and all-round functional exercises of the shoulder joint were carried out gradually. Regular reexaminations of anteroposterior and lateral X-ray films of the shoulder joint were conducted postoperatively on day 1, and at 1, 3, 6, and 12 months after the operation to further evaluate the fracture healing. Humeral head height loss and the neck-shaft angle were measured on postoperative and follow-up radiographs on X-ray films immediately after the operation and at each follow-up time. Intraoperative blood loss was estimated by the anesthesiologist based on the volume in the suction canister after subtracting the amount of irrigation fluid and by weighing blood-soaked gauze pads.

Outcome assessments at follow-up visits were performed independently by two senior orthopedic surgeons. Visual Analog Scale (VAS) for pain, American Society of shoulder and Elbow Surgeons (ASES) Score, and Disabilities of the Arm, Shoulder and Hand (DASH) score and the Constant–Murley score for shoulder joint function were respectively evaluated and recorded for the function and pain of the affected shoulder joint, and the complications were also recorded.

The primary outcome measures of this study were the neck-shaft angle, height of humeral head loss, and functional outcomes (VAS, ASES, DASH, and Constant–Murley scores) at the last follow-up. Secondary outcomes included length of hospital stay, fracture healing time, intraoperative blood loss, and the incidence of postoperative complications.

### Statistical analysis

Data analysis was performed using IBM SPSS Statistics software (version 28.0; IBM Corp). For measurement data, the Shapiro–Wilk test was first used to determine whether they were normally distributed. Continuous variables are presented as mean ± standard deviation (SD) if normally distributed. One-way analysis of variance (ANOVA) was used for comparisons among the three groups. *Post-hoc* pairwise comparisons were performed using the Tukey test (or Bonferroni correction). χ^2^ test was used for comparisons between groups of count data. A *P* value < 0.05 was considered statistically significant.

## Results

This study was a retrospective cohort study, with a total of 51 patients included, including 16 males and 35 females. Age: Mean age was 62.4 ± 10.2 years (range, 59–78 years). Causes of injury: 40 cases of fall injuries and 11 cases of traffic injuries. There was no statistically significant difference in the general data such as gender, age, injured side, and Neer classification among the three groups of patients (*P* > 0.05), and they were comparable (Table 1).

**Table 1:** Baseline characteristics of patients with proximal humeral fractures and medial column support loss.

Variable	Double-board group (*n* = 17)	Bone-grafting group (*n* = 17)	Steel plate group (*n* = 17)	*F*/χ^2^	*P*-value
Demographic characteristics					
Age (years, mean ± SD)	65.6 ± 12.2	63.1 ± 10.5	60.6 ± 11.2	1.212	0.602
Sex (Male/Female, n)	6/11	5/12	5/12	0.182	0.935
BMI (kg/m^2^, mean ± SD)	25.3 ± 3.1	24.8 ± 3.4	25.1 ± 3.0	0.215	0.807
Fracture characteristics					
Side (Left/Right, n)	8/9	7/10	9/8	0.472	0.790
Neer classification (III/IV, *n*)	12/5	11/6	12/5	0.182	0.913
Combined dislocation [*n* (%)]	4 (23.5)	5 (29.4)	4 (23.5)	–	1.000
Involvement of anatomical neck [*n* (%)]	5 (29.4)	6 (35.3)	5 (29.4)	0.182	0.913
Bone quality					
Bone mineral density *T*-score (mean ± SD)	−2.9 ± 0.5	−3.0 ± 0.6	−2.8 ± 0.5	0.672	0.515
Comorbidities					
Hypertension [*n* (%)]	8 (47.1)	7 (41.2)	8 (47.1)	0.158	0.924
Diabetes mellitus [*n* (%)]	4 (23.5)	5 (29.4)	4 (23.5)	–	1.000

The blood loss amounts of the three groups of patients were 450.6 ± 13.7 ml, 380.5 ± 15.3 ml, and 280.2 ± 10.3 ml respectively. The double plate group had the most, the bone graft group was in the middle, and the plate group had the least. Pair-to-pair-to-plate comparisons showed statistically significant differences (*P* < 0.05). There was no statistically significant difference in the length of hospital stay and fracture healing time among the three groups of patients (*P* > 0.05) (Table 2).

**Table 2:** Operative data and fracture healing time.

Variable	Double-board group (*n* = 17)	Bone-grafting group (*n* = 17)	Steel plate group (*n* = 17)	*F*-value	*P*-value
Hospital stay (*d*)	16.5 ± 2.4	15.8 ± 2.6	15.1 ± 1.8	3.392	1.531
Intraoperative blood loss (ml)	450.6 ± 13.7	380.5 ± 15.3^#^	280.2 ± 10.3^*^^#^	2.246	< 0.05
Fracture-healing time (*w*)	6.6 ± 1.3	5.9 ± 1.0	6.1 ± 1.2	1.489	0.532

^#^Compared with the double-plate group < 0.05,

^*^compared with the bone graft group < 0.05.

The cervical trunk angles of the three groups of patients at the last follow-up were 132.1° ± 9.8°, 125.3° ± 5.4°, and 120.1° ± 7.5° respectively. The cervical trunk Angle in the double-plate group increased significantly, and the differences between any two groups were statistically significant (*P* < 0.05). At the last follow-up, the heights of humeral head loss were 1.32 mm ± 0.33 mm, 2.04 mm ± 0.56 mm, and 3.12 mm ± 0.85 mm respectively. The height of humeral head loss in the double-plate group was significantly lower than that in the other two groups, and the differences between each pair were statistically significant (*P* < 0.05). At the last follow-up, the range of motion of shoulder joint forward flexion was 158.6° ± 12.8°, 149.3° ± 10.5°, and 140.2° ± 11.3°, respectively. The degrees of abduction range of motion were 148.2°±10.4°, 140.3° ± 11.5°, and 134.3° ± 12.6° respectively. The degrees of forward flexion and abduction range of motion in the double-plate group were significantly better than those in the other two groups, and the differences between each pair were statistically significant (*P* < 0.05). The external rotation range of motion was 41.2° ± 7.8°, 40.5° ± 6.1°, and 39.8° ± 7.4° respectively, and the posterior extension range of motion was 36.6° ± 1.2°, 35.8° ± 1.0°, and 35.2° ± 1.4° respectively. There were no statistically significant differences among the three groups (*P* > 0.05) (Table 3).

**Table 3:** Operative data and fracture healing time.

Variable	Double-board group (*n* = 17)	Bone-grafting group (*n* = 17)	Steel plate group (*n* = 17)	*F*-value	*P*-value
Humeral neck angle (°)	132.1 ± 9.8	125.3 ± 5.4^#^	120.1 ± 7.5^#^^*^	0.318	< 0.05
Head loss height (mm)	1.32 ± 0.33	2.04 ± 0.56^#^	3.12 ± 0.85^*^	2.645	< 0.05
Forward flexion range of motion (°)	158.6 ± 12.8	149.3 ± 10.5^#^	140.2 ± 11.3^#^^*^	4.712	< 0.05
Outreach activity level (°)	148.2 ± 10.4	140.3 ± 11.5^#^	134.3 ± 12.6^#^^*^	3.986	< 0.05
External rotation activity (°)	41.2 ± 7.8	40.5 ± 6.1	39.8 ± 7.4	2.435	0.074
Posterior range of motion (°)	36.6 ± 1.2	35.8 ± 1.0	35.2 ± 1.4	1.627	0.034

^#^Compared with the double-plate group < 0.05,

^*^compared with the bone graft group < 0.05

**Figure 2:**
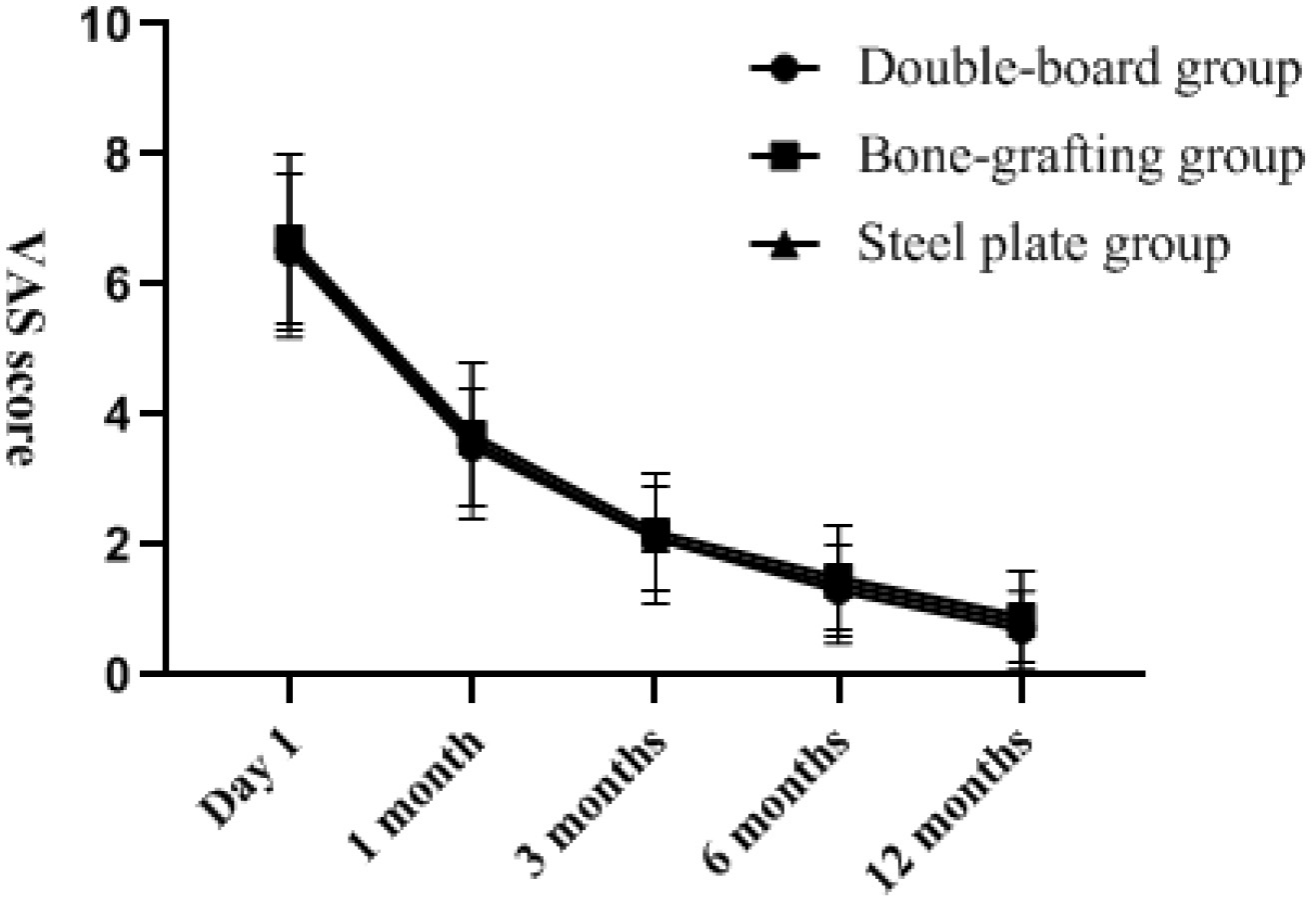
Visual Analogue Scale for Pain on the 1st day of postoperative follow-up, and at the 1st, 3rd, 6th, and 12th months. The pain was significantly relieved with the extension of the follow-up time.

**Figure 3:**
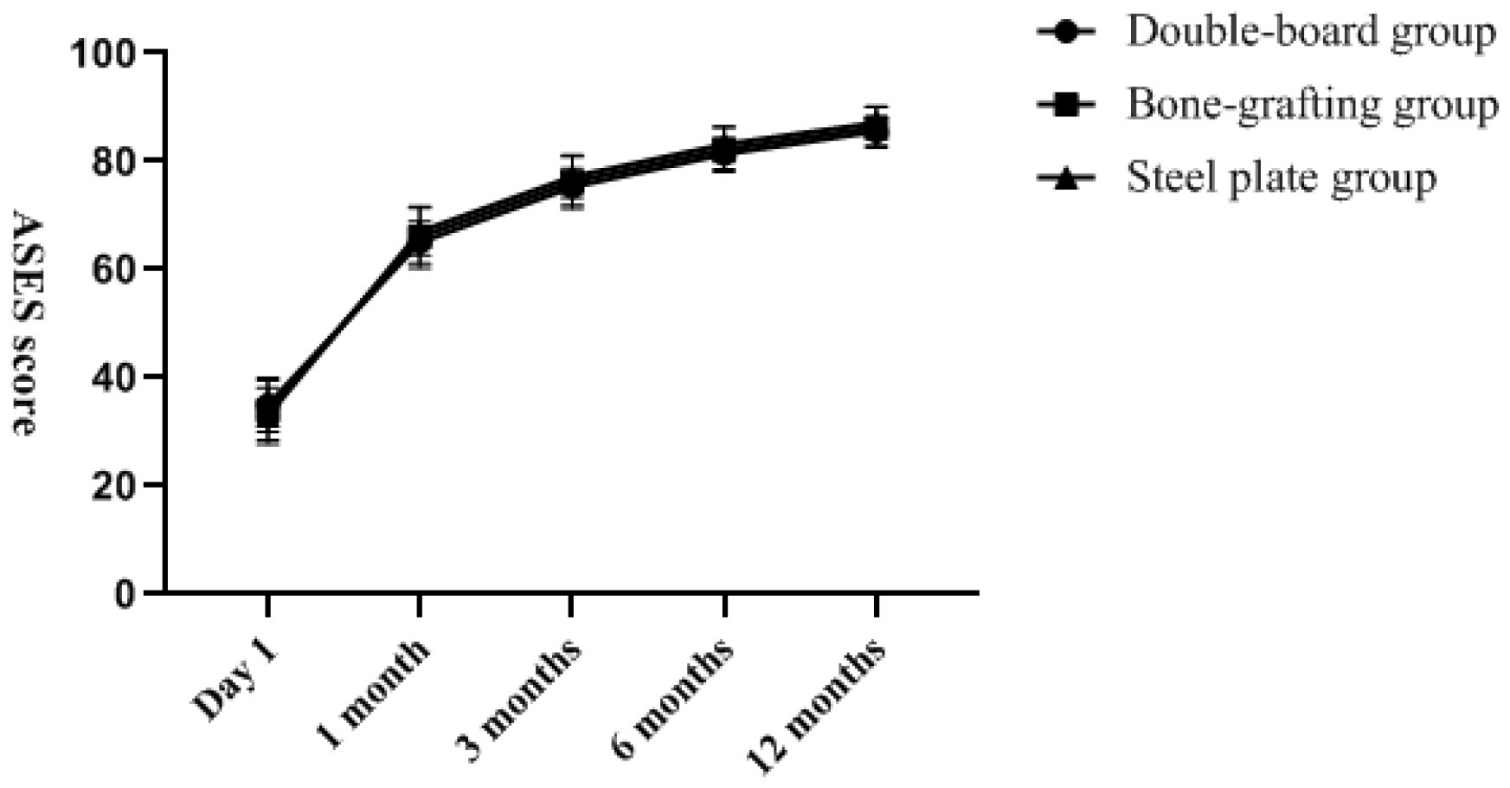
The American Society of Shoulder and Elbow Surgeons Scoring Scales on the first day of postoperative follow-up, at the 1st, 3rd, 6th, and 12th months, and the scores became better and better with the extension of the follow-up time.

**Figure 4:**
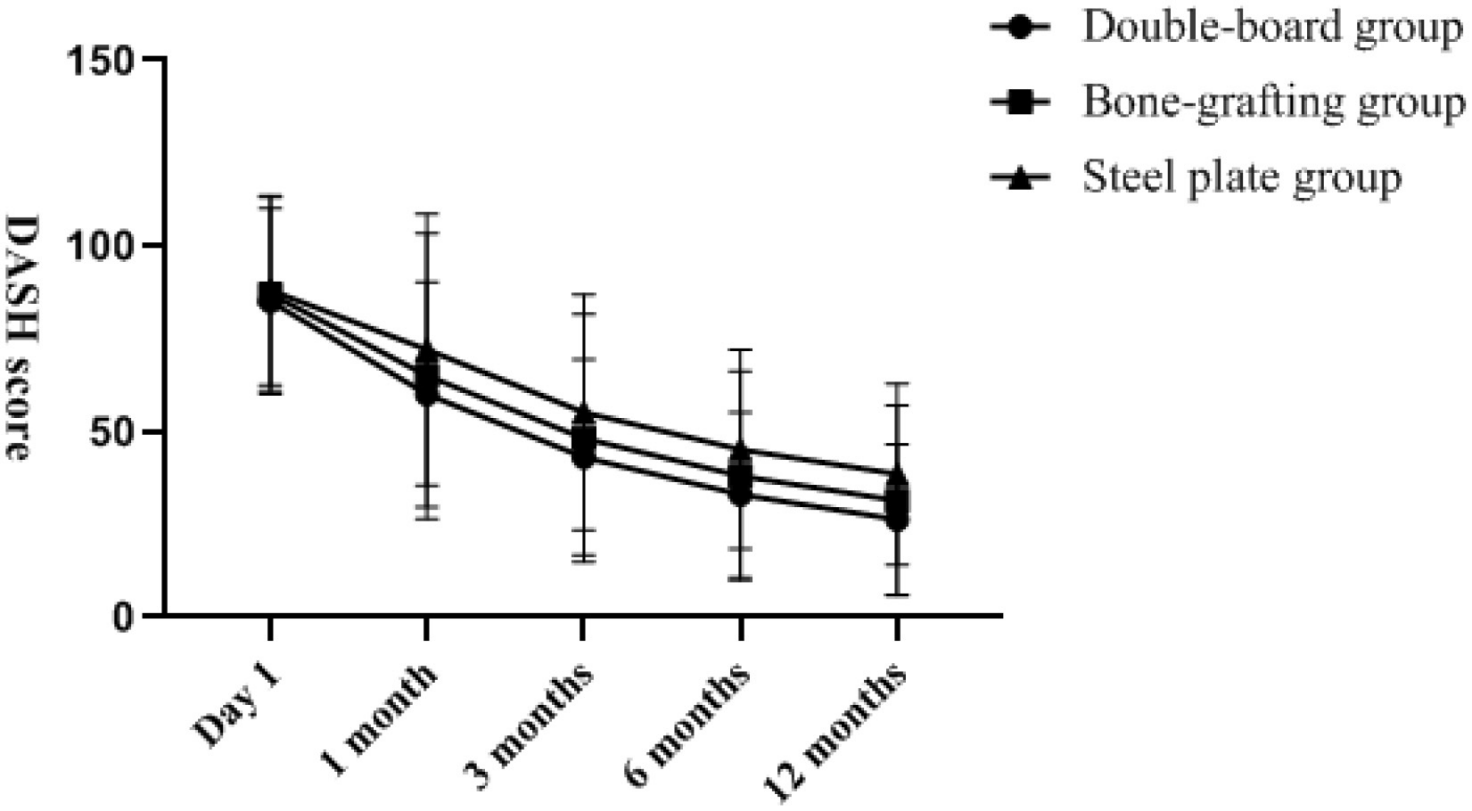
There was no significant difference in the postoperative DASH score among the three groups.

**Figure 5:**
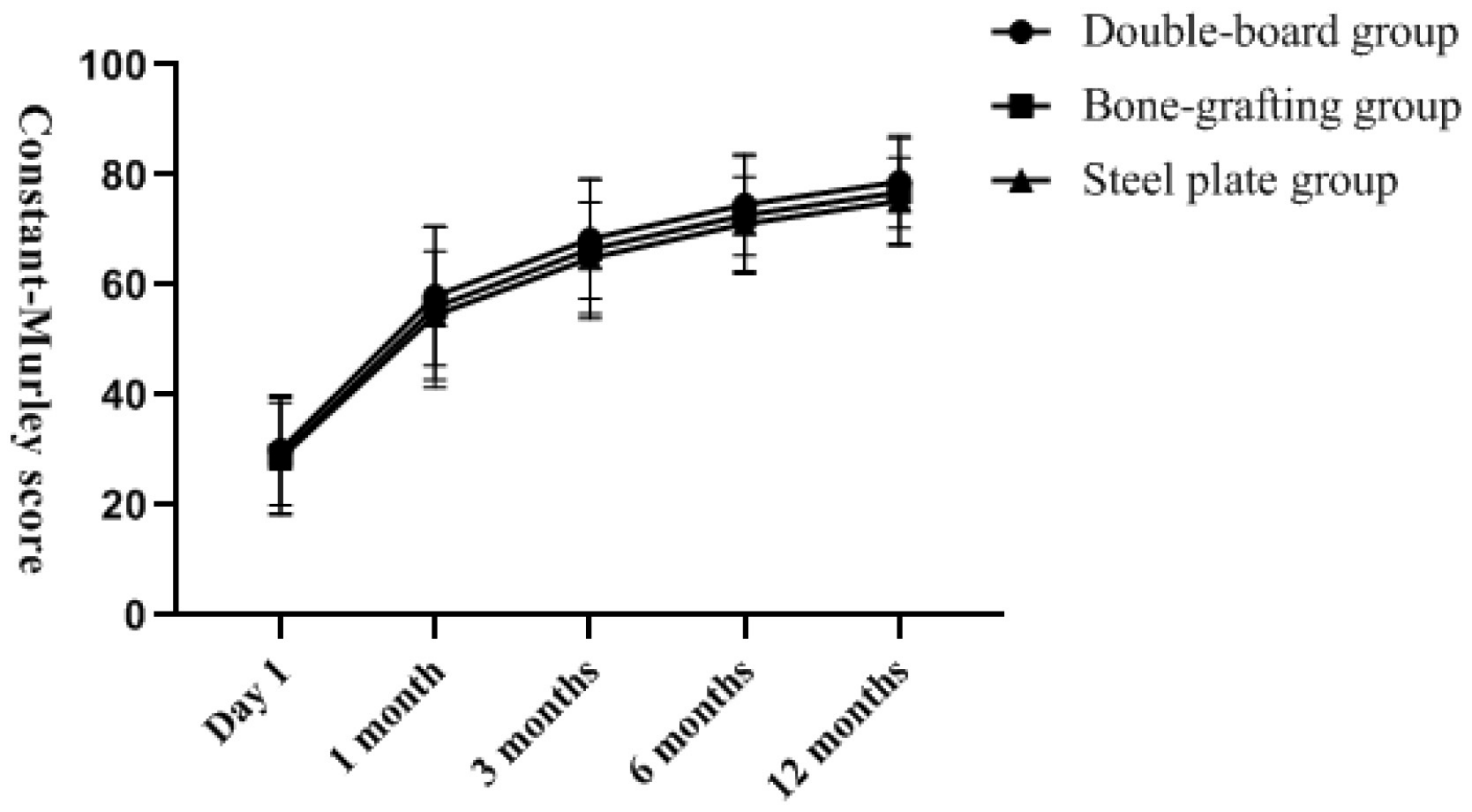
Postoperative Constant-Murley scores among the three groups. With the extension of time, the functional scores gradually thickened, and there was no significant difference among the three groups.

There was no statistically significant difference in VAS score, ASES score, DASH score and Constant–Murley score among the three groups of patients at the last follow-up (*P* > 0.05). The changes of VAS score, ASES score, DASH score, and Constant-Murley score with follow-up time are shown in Figures 2–5. Complications: shoulder joint impact syndrome occurred in one case of the double-plate group; Shoulder joint stiffness occurred in 2 cases of the bone grafting group. In the steel plate group, there was 1 case of screw loosening and 2 cases of screw resection. There was no statistically significant difference in the number of complications among the three groups (*P* > 0.05), as shown in Table 4.

**Table 4:** Patient-reported outcome measures at final follow-up.

Variable	Double-board group (*n* = 17)	Bone-grafting group (*n* = 17)	Steel plate group (*n* = 17)	*F*/χ^2^	*P*-value
VAS score	0.7 ± 0.6	0.9 ± 0.7	0.8 ± 0.8	0.186	0.766
ASES score	85.5 ± 2.6	86.3 ± 3.8	87.1 ± 2.9	0.426	0.502
DASH score	26.25 ± 20.21	31.38 ± 25.62	38.47 ± 24.36	0.341	0.656
Constant–Murley score	78.8 ± 8.4	76.9 ± 9.7	75.3 ± 7.8	0.752	0.581
Total complications [*n* (%)]	1 (5.9)	2 (11.8)	3 (17.6)	–	0.860

VAS stands for Visual Analog Scale. ASES scores the American Society of Shoulder and Elbow Surgeons; DASH scores life skills; Constant–Murley scored the function of the shoulder joint.

## Discussion

This study compared three reconstruction strategies for medial column support loss in elderly patients with proximal humeral fractures: dual plating, locking plate combined with autogenous iliac crest bone grafting, and a single lateral locking plate. We found that the dual plating technique was more effective in maintaining the neck-shaft angle and preventing humeral head height loss, and it provided better shoulder flexion and abduction range of motion at the final follow-up. However, there were no significant differences among the three groups in VAS, ASES, DASH, or Constant–Murley scores, and the overall incidence of complications was low and comparable across groups.

China has a huge population base. Population aging is an important trend in social development. Due to the current changes in population structure, the number of proximal humeral fractures has risen sharply. This is also the basic national condition of China for a relatively long period of time in the future (8). Locking plate is currently the most widely used surgical method for treating proximal humeral fractures. Based on the force characteristics of the shoulder joint, the lateral plate forms a force structure similar to that of a crane arm, which inevitably leads to insufficient support on the medial side of the fracture. Since the medial column of the proximal humerus is a transitional area between cancellous bone and cortical bone, the trabecular bone is weak and belongs to a mechanically weak area. Fractures in this specific area may lead to compression or fracture of the medial column. Treatment with the lateral locking plate alone usually results in loss of the cervical trunk Angle, screw ejection, varus deformity, and even ischemic necrosis of the humeral head. According to statistics, the incidence of postoperative complications is as high as 49% (9). In recent years, the literature has reported that the medial column can enhance the stability of internal fixation. However, there are still many deficiencies in the current research and application. The absence of the medial column is one of the important factors causing implantation failure and complications. It has outstanding performances in biomechanics such as maintaining the height of the humeral head to prevent inversion and increasing the stiffness, anti-rotational force, and anti-shear force of the locking plate. It can maintain the stability of internal fixation and reduce postoperative complications (10, 11). Therefore, the treatment of proximal humeral fractures remains challenging. When surgical fixation is decided upon, anatomical reduction and restoration of medial support, as well as protection of vascular and periosteal structures, are key prognostic factors and the most reliable features for preventing secondary varus displacement.

When a proximal humeral fracture involves the medial column, it is usually accompanied by the destruction of the medial column support structure. In severe cases of fractures, bone defects of the medial support structure may occur, which will inevitably affect the stability of the proximal humeral fracture after reduction. Therefore, it is necessary to repair and reconstruct the stability of the medial column support to reduce related complications and improve the therapeutic effect.

Regarding the choice of materials for medial column reconstruction, commonly used augmentation options include autologous iliac crest bone grafts, autologous or allogeneic fibular strut grafts, and various synthetic bone substitutes. Multiple clinical and biomechanical studies have demonstrated that these materials can improve the stability of internal fixation for proximal humeral fractures in the setting of osteoporosis to varying degrees (6, 7, 12–15). Previous studies have shown that autologous iliac crest bone grafts provide excellent osteogenic potential and simultaneously offer both cortical and cancellous bone, thus affording reliable biological and mechanical support for medial column defects in elderly osteoporotic patients (10, 12). In contrast, allogeneic fibular strut grafts exhibit strong structural support in restoring medial column integrity and preventing varus collapse and have become one of the most widely used augmentation techniques in the current literature; however, their application is limited by higher costs, restricted donor availability, and the potential risks of immune reaction and infection (7, 13, 15). Taking into account patient economic burden, material availability, and the technical and resource conditions of local medical institutions, autologous iliac crest bone grafting was adopted as one of the medial column reconstruction methods in this study, which is more consistent with the current practice patterns of most trauma centers in China.

One of the relatively mature and most widely used methods in clinical practice is intramedullary support, which involves placing a structural internal fixation device in the medullary cavity of the humeral head to indirectly support and reconstruct the medial column, significantly enhancing the stability of the fixation. Commonly used grafts for intramedullary support include allogeneic fibula, autologous fibula, and autologous iliac bone. Allogeneic fibula support surgery takes longer and is more expensive, with risks such as infection due to inadequate sterilization, which may lead to legal restrictions in some countries and prevent its use. The fibula, as a lateral support of the lower limb, bears approximately 1/6 of the load. The proximal end of the fibula articulates with the lateral side of the tibia, forming the superior tibiofibular joint, and the distal end participates in the formation of the ankle joint as the lateral malleolus. The integrity of the fibula has a significant impact on the stability of the ankle joint. Secondly, the destruction of the integrity of the fibula is bound to affect the functions of the hip, knee, and ankle joints. Iliac bone grafting has more advantages than fibular grafting. Iliac bone grafting has the advantages of being easier to obtain than bones from other parts, having less impact on the donor site, being superficial at the bone harvesting site, having no important blood vessels or nerves passing through, no risk of rejection, strong osteoinductive effect, and the ability to shape the iliac bone with bone forceps.

At present, there is no unified standard protocol for reconstructing the medial column of the proximal humerus. Zhu et al. (12) compared the treatment of four-part fractures of the proximal humerus using locking plates combined with autologous iliac bone marrow graft to enhance medial support with fractures treated without any form of reinforcing plates. The results of the locking plate combined with autologous iliac bone group showed faster fracture healing, higher postoperative function and quality of life. Even so, autologous bone transplantation still has some drawbacks such as limited sources and donor site complications. Gardner et al. (13) first utilized allogeneic fibula, placed it within the bone and combined it with a locking screw structure to assist in reduction and restoration of the mechanical integrity of the medial column of the proximal humerus. However, they also mentioned issues such as the high cost of using this method and the increased risk of infection. Subsequent studies have found that humeral distance screws can support the medial column and improve the stability of proximal humeral fixation. However, their supporting effect is limited, especially for patients with medial column crushing and poor medial column reduction. Although simple humeral distance screw fixation reconstructs the “beam” structure of the medial column, the defect of the medial column bone still exists. Moreover, in patients with osteoporosis, it is easy for the screws to have weak grip. The humeral talus screws have difficulty achieving a tight combination with the inner and lower part of the humeral head and cannot provide additional support effects (14, 15).

Therefore, clinicians and a large number of researchers have begun to conduct research and exploration on the reconstruction of extramedullary supports in the proximal humerus. Currently, the most common method of extramedullary support reconstruction in clinical practice is the fixation of the medial cortical bone with additional plates (16, 17). He et al. directly supported the humeral head by placing an anatomical plate on the medial side of the humeral head, forming an internal and lateral “double-column support” structure with the lateral locking plate, thereby achieving the stability of proximal humeral fractures (18). The results of the finite element analysis of biomechanics show that the mechanical properties of this enhanced fixation method are superior to those of the simple locking steel plate, the lateral locking plate combined with the intramedullary fibula support, and the lateral locking plate combined with the posterior side steel plate, At the same time, it also solves the stress concentration problem caused by the boom structure of the lateral locking plate itself (19). Research shows that the key to improving the stress distribution of the internal fixation device with double steel plates lies in the reconstruction of the medial column support. The medial auxiliary reconstruction steel plates play the role of bridging and supporting, which helps to resist the continuous varus and valgus stress generated during rotator cuff contraction. Moreover, the reconstructed medial column support further dispersions the stress between the screw and the bone interface, providing mechanical support for the humeral head. It can also effectively correct the fracture position. More importantly, it can improve the stability of internal fixation, promote the rapid healing of fractures, and further improve the function and range of motion of the shoulder joint (20). The research of Wang et al. (21) shows that compared with the use of locking plates alone, the use of locking plates combined with medial support in the treatment of comminuted fractures of the proximal humerus in the elderly can reduce complications such as inversion and displacement, promote the revascularization of the humeral head, and accelerate fracture healing. diTullio et al. (22) tested the biomechanical effect of fixation with two plates using a proximal humeral fracture model. The results showed that the combination of the lateral locking plate and the medial non-locking plate was the most robust and could provide a more stable fixation effect. Although the fixation was more stable, there was a risk of damaging the axillary nerve and the rotator brachial vessels, and the soft tissue dissection was extensive. The local blood supply is severely damaged, and the risk of bone nonunion is high. The use of inner steel plates is currently limited to biomechanical research, and there are relatively few clinical studies. According to the usage principles of AO internal fixation: Internal fixators should not be placed on the stress side to minimize damage to blood supply. However, the medial support plate is exactly the opposite. The medial side is not easily exposed, and more medial structures need to be stripped, which disrupts blood supply and poses a higher surgical risk. This might be the reason why the medial plate for proximal humeral fractures cannot be clinically promoted. Katthagen et al. (23) hold that the auxiliary application of anterior medial support plates in biomechanics is of great help for fracture reduction and fixation, especially in cases of poor bone quality and obvious medial instability (such as fractures involving lesser tuberositys, varus fractures with medial column defects, and comminuting fractures of the metaphysis). To reduce soft tissue dissection, axillary nerve and rotator brachial vascular injury, we placed the support plate on the anterior medial side instead of the medial side, which could effectively avoid damaging the ischemic necrosis of the humeral head and the blood supply of the medial rotator brachial artery. This fixation method has clinical significance in the surgical treatment of medial column absence in proximal humeral fractures and is also in line with biomechanical advantages.

At the final follow-up, there were no statistically significant differences in VAS, ASES, DASH, or Constant–Murley scores among the three groups. Notably, the relatively large standard deviations observed in the DASH scores suggest substantial individual variability in upper limb functional recovery among patients. This finding may be attributed, on one hand, to the inclusion of an elderly, osteoporotic population with multiple chronic comorbidities, as well as considerable heterogeneity in activities of daily living and rehabilitation adherence, which may contribute to the discrepancy between subjective functional perception and objective functional performance. On the other hand, the absence of stratified analyses based on socially relevant factors such as occupational demands, dominance of the affected upper limb, and living environment may have further amplified the dispersion of DASH scores (4, 6, 11).

This study has several limitations: Firstly, due to the inherent limitations of retrospective studies, despite setting relatively strict inclusion and exclusion criteria, there is still a possibility of selection bias and confounding. Secondly, due to the short follow-up period, small sample size, and being a single-center study, further prospective, multi-center, large-sample, and complete long-term follow-up studies are still needed for verification. Finally, we were unable to compare the outcomes between additional surgeries (such as medial malleolar screws and fibular grafts), and further comparative studies are required.

## Conclusion

In conclusion, this retrospective study comparing three techniques for reconstructing medial column support loss in elderly patients with proximal humeral fractures found that dual plating (lateral locking plate combined with an anteromedial support plate) was associated with superior radiographic outcomes (higher neck-shaft angle, less humeral head height loss) and better shoulder range of motion (forward flexion and abduction) compared to locking plate fixation with autologous iliac bone graft or locking plate fixation alone. Functional outcome scores and complication rates, while not statistically different, showed a trend favoring the dual plating group. However, the dual plating technique resulted in significantly greater intraoperative blood loss. Therefore, while dual plating demonstrates advantages in restoring and maintaining medial column stability and early functional recovery, its use should be balanced against the increased surgical invasiveness. The choice of reconstruction technique should be individualized based on patient factors, fracture characteristics, and surgeon expertise.

## Data Availability

The datasets presented in this study can be found in online repositories. The names of the repository/repositories and accession number(s) can be found in the article/supplementary material.
